# Identification of a Novel *COL4A4* Variant in Compound-Heterozygous State in a Patient With Alport Syndrome and Histological Findings Similar to Focal Segmental Glomerulosclerosis (FSGS)

**DOI:** 10.3389/fgene.2018.00748

**Published:** 2019-01-28

**Authors:** Feng Zhu, Wencheng Li, Zhenqiong Li, Hongyan Zhu, Jing Xiong

**Affiliations:** ^1^Department of Cardiology, Union Hospital, Tongji Medical College, Huazhong University of Science and Technology, Wuhan, China; ^2^Department of Urology, Union Hospital, Tongji Medical College, Huazhong University of Science and Technology, Wuhan, China; ^3^Department of Nephrology, Union Hospital, Tongji Medical College, Huazhong University of Science and Technology, Wuhan, China

**Keywords:** Alport syndrome, *COL4A4*, focal segmental glomerulosclerosis, targeted next generation sequencing, novel variants, genetic counseling

## Abstract

Alport syndrome (AS) is a rare and inherited renal disorder with an autosomal recessive mode of inheritance. AS patients usually manifest with hematuria and progressive renal disorder also occasionally accompanied by hearing loss and ophthalmic disease. Germline variants in collagen type IV α-4 (*COL4A4*) gene lead to autosomal recessive Alport syndrome. In the present study, we investigated a Chinese family with Alport syndrome. The index patient is a 24-year-old Chinese woman who has been suffering from proteinuria. Renal biopsy and renal pathology were performed and found focal segmental glomerulosclerosis (FSGS) like lesion in the index patient. The index patient also presented with binocular edema and blurred vision. However, binocular edema dissipated gradually without any further treatment. Unlikely, the index patient was not diagnosed with hearing impairment. Index patient’s parents are phenotypically normal. Targeted next generation sequencing and Sanger sequencing was performed. A novel heterozygous single nucleotide insertion, c.4760_4761insC and a previously reported likely pathogenic variant, c.1323_1340delTGGCTTGCCTGGAGCACC in the *COL4A4* gene were identified in the index patient. The novel heterozygous single nucleotide insertion (c.4760_4761insC) leads to a frameshift which eventually results in the formations of a truncated COL4A4 protein. In addition, the other heterozygous likely pathogenic variant, c.1323_1340delTGGCTTGCCTGGAGCACC, has been already identified with causing AS an autosomal recessive mode of inheritance. Sanger sequencing confirmed that these two variants were inherited in the index patient from her father and mother, respectively. These two variants were not found in 100 normal control individuals. In conclusion, our present finding emphasizes the significance of high throughput targeted next generation sequencing technology for rapid and cost-effective genetic screening which allows us easy and accurate clinical diagnosis of AS patients.

## Introduction

Alport syndrome (AS) is a rare and hereditary renal disorder. AS is characterized by the progressive loss of renal function accompanied with hematuria and proteinuria. Usually AS patients present sensorineural hearing impairment, and lenticonous and macular flecks (Mochizuki et al., 1994). According to the previously reported cases, AS is majorly (85%) inherited with a X-linked dominant manner while, and autosomal recessive AS (ARAS) is extremely rare (15%) (Boye et al., 1998; Pierides et al., 2013). Germline variants in Collagen α-4 (IV) chain (*COL4A4*) and Collagen α-3 (IV) chain (*COL4A3*) gene cause ARAS (Hanson et al., 2011). However, ARAS affects individuals irrespective of gender (Heidet et al., 2001). *COL4A4* associated ARAS is a very rare disorder which is usually reported with extreme phenotypic heterogeneity (Marcocci et al., 2009).

*COL4A4* gene is located in chromosome 2 and encodes the α4 chains of type IV collagen. Glomerular basement membrane (GBM) is primarily composed of type IV collagen. Variants in *COL4A4* gene lead to the formation of abnormal or non-functional type IV collagen which eventually results into Alport syndrome (AS) [MIM# 203780] or thin basement membrane nephropathy [MIM# 141200] (Deltas et al., 2013).

In patients with Alport syndrome, molecular genetic diagnosis is usually performed by Sanger sequencing of three genes, namely, *COL4A3*, *COL4A4* and *COL4A5*. These three genes together comprise of 153 exons, so performing Sanger sequencing is very laborious, time consuming and not a cost-effective approach for identifying the candidate variants. Recent development in high-throughput sequencing technology allows us to perform a rapid, accurate, cost-effective approach to identifying the candidate variants in these three genes by targeted next generation sequencing (Morinière et al., 2014; Xiu et al., 2014).

Here, we identified a 24-year-old Chinese woman with proteinuria. Focal segmental glomerulosclerosis (FSGS) like lesion has been identified by renal biopsy and renal pathology. She also clinically manifested with binocular edema and blurred vision but without any symptoms of hearing impairment. Index patient’s parents are phenotypically normal. Targeted next generation sequencing identified a novel heterozygous single nucleotide insertion, c.4760_4761insC and a previously reported likely pathogenic variants, c.1323_1340delTGGCTTGCCTGGAGCACC in the *COL4A4* gene in the index patient (Boye et al., 1998; Nabais Sá et al., 2015; Imafuku et al., 2018).

Our present study not only expands the mutational spectrum for the *COL4A4* gene associated with ARAS in this family, but also highlights the significance of targeted next generation sequencing for identifying candidate variants in patients with ARAS.

## Case Report

**FIGURE 1:**
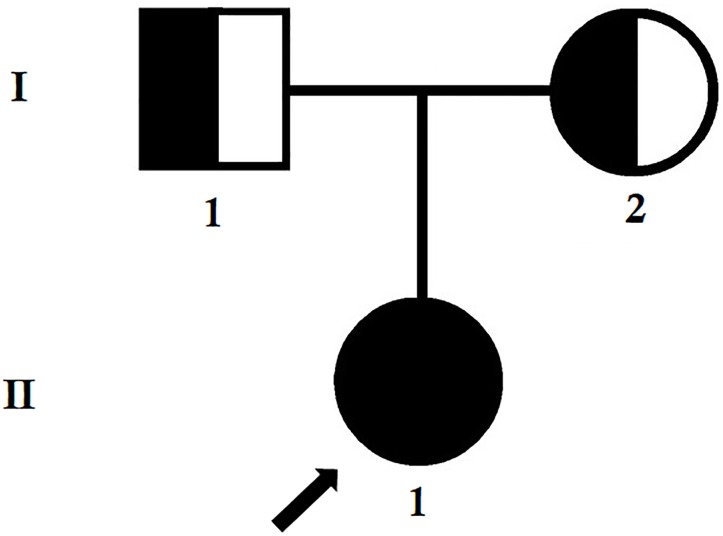
Pedigree of the family. The filled symbol indicates the patient (index patient), and the half-filled symbols show the carrier parents, who were heterozygous carriers but were asymptomatic. The arrow points to the index patient.

The index patient was a 24-year-old Chinese woman from non-consanguineous parents (Figure 1). The index patient was healthy on birth. Since the age of 16 years, the index patient has been suffering from mild proteinuria with normal level of serum creatinine (the normal range of creatinine is 44–106 μmol/L for female). No special treatment was recommended and only periodic review was performed. At the age of 20 years, the index patient gradually developed proteinuria which was occasionally accompanied with binocular edema and blurred vision. Angiotensin converting enzyme inhibitors (ACEI) and some traditional Chinese medicine were recommended for the patient, but the result was not satisfactory. Traditional Chinese medications (for example, Shenyan Kangfu tablet, Huangkui capsule) were used to reduce the proteinuria. Gradually, proteinuria and edema became more serious, so the patient was admitted to our hospital to perform further examination at the age of 24 years.

Pathological tests and routine blood tests of the index patient showed the following results: albumin 38.1 g/L (35–55 g/L), triglyceride 2.31 mmol/L (<1.7 mmol/L), HDL-C 2.18 mmol/L (1.29–1.55 mmol/L), LDL-C 2.26 mmol/L (2.7–3.1 mmol/L) and creatinine 54.2 μmol/L.

There was no abnormality in antinuclear antibody (ANA), antineutrophil cytoplasmic antibodies (ANCA), hepatitis B and free light chain. Urine routine test found proteinuria and erythrocyturia, without leukocyturia. Erythrocyturia manifested with dysmorphic erythrocytes, and 24-h quantitative urine protein was 5.067 g. In the index patient, urine protein screening found that the patient has been suffering from non-selective proteinuria. Albumin creatinine ratio (ACR) was 3200 mg/g. High frequency hearing loss was found by further examination of the index patient, without ocular lesion. FSGS like lesion were identified by renal biopsy and renal pathology. In addition, electron microscopic study revealed uneven thickening of GBM with splitting of the lamina densa. Hence, we suspected that the index patient may be suffering from Alport syndrome. Parents of the index patient and other members of this family were completely normal.

### Light Microscopy (LM) Examination

**FIGURE 2:**
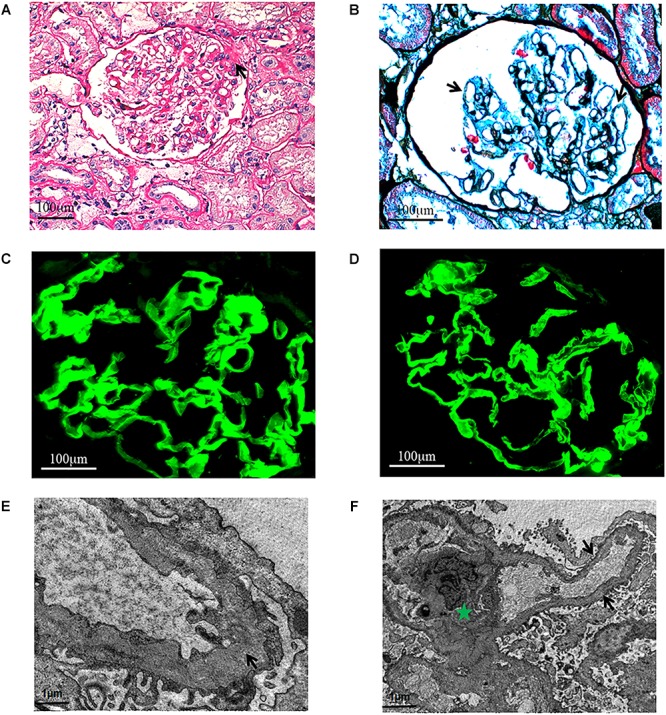
Light microscopy. **(A)** Focal segmental glomeruosclerosis (x400, PAS.). One segmental sclerosis with top adhesion (black arrow). **(B)** Diffuse vacuolar degeneration (black arrows) of the GBM (x600, PASM+Masson.). Immunofluorescence. The α3 and α5 chains are distributed in the GBM (x600). **(C)** α3 chain. **(D)** α5 chain. Electron microscopy. **(E)** The thickness of GBM is different and the density is uneven (black arrow). Green Pentagram shows mesangium and mesangial cells. **(F)** The mesangial insertion can be seen in a few of segments, and the dense layer of GBM is tearing and stratified (black arrow).

Light microscopy study found FSGS (Figure 2A) and diffuse vacuolar degeneration of the GBM in the index patient (Figure 2B). Tubular epithelial cells were swollen or granulated, and scattered foam cells were found in the interstitium.

### Immunofluorescence Examination

Immunofluorescence study revealed that the α3 and α5 collagen IV chains were well distributed in the GBM (Figure 2C,D) and IgG, IgM, IgA, C3, C1q, and C4 staining were negative. No immunoglobulin and complement components were deposited outside the glomerulus.

### Electron Microscopy (EM) Examination

Electron microscopic examination found that the thickness of GBM was not regular and the density was also uneven. The dense layer of GBM was tearing and stratified. Mesangial insertion could be seen in few segments (Figure 2E,F). There was diffuse effacement of foot processes.

Based on the clinical symptoms, the index patient and her parents were recommended for performing molecular genetic diagnosis by targeted next generation sequencing and confirmatory Sanger sequencing.

## Materials and Methods

### Patients and Pedigree

The index patient was clinically diagnosed with AS and treated in the Department of Nephrology, Union Hospital, Tongji Medical College, Huazhong University of Science and Technology, Wuhan, China. Clinical diagnosis of AS in the index patient has been done based on the clinical symptoms, laboratory tests, renal biopsy and pathological examinations.

### Kidney Pathology Evaluation

Percutaneous renal biopsy was performed under ultrasonographic guidance. Formalin-fixed tissue was embedded in paraffin using routine procedures. Sections of 1 μm in thickness were stained with haematoxylin and eosin (HE), periodic acid-Schiff (PAS), periodic acid-methenamine silver (PAMS), Masson’s trichrome-elastica and Congo red for LM pathological diagnosis. Immunofluorescence staining was performed on 3 μm cryostat sections by using fluorescein isothiocyanate-(FITC) labeled rabbit anti-human immunoglobulin (Ig) G, IgA, IgM, complement (C) 3, C4, C1q, α3, and α5 collagen IV chains antibodies (DAKO Denmark A/S, Denmark).

Transmission electron microscopy was performed to observe the ultra-microstructures of the renal specimen which was cut into ultra-thin sections of 60–68 nm.

### Targeted Next-Generation Sequencing and Variant Identification

Genomic DNA samples obtained from the index patient (II-1) were sequenced using targeted next-generation sequencing. Roche NimbleGen’s (Madison, WI, United States) custom Sequence Capture Human Array was designed to capture 15162 kb of targeted sequence, covering 153 exons and 100 bp, of flanking introns of 3 genes (*COL4A4, COL4A3,* and *COL4A5*) which are associated with AS. The average sequencing depth of the target area is 241.04 with 100% coverage. The procedure for preparation of libraries was consistent with standard operating protocols published previously (Yang et al., 2018).

### Sanger Sequencing

To validate putative variants, Sanger sequencing was performed. Primers flanking the candidate loci were designed based on the reference genomic sequences of the Human Genome from GenBank in NCBI and synthesized by Invitrogen, Shanghai, China. The heterozygous variants identified through targeted next generation sequencing were verified through Sanger sequencing using the following primers: F1 5-ACCCCATGACGCTTAATCAGGC-3, R1 5-GCAGCTTCTCACCCGGTTCTG-3 and F2 5-AGCGCATGTCGATTAATGCGAC-3, R2 5-ACAGGTAACTTCAAGCCTTAACAG-3. The reference sequence NM_000092 of *COL4A4* was used.

### Identification of Novel Variants

**FIGURE 3:**
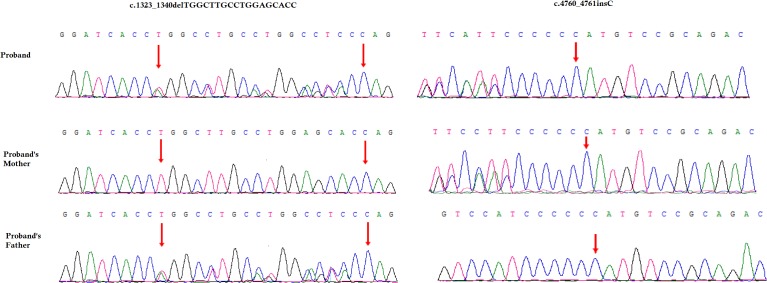
Partial DNA sequences in the *COL4A4* by Sanger sequencing of the family. Upper line: the index patient, middle line: the mother, bottom line: the father. Arrows point to the variants. The index patient inherited both c.4760_4761insC and 18 bp deletion (c.1323_1340delTGGCTTGCCTGGAGCACC) variants. The mother carries the c.4760_4761insC variants and the father carries 18 bp deletion (c.1323_1340delTGGCTTGCCTGGAGCACC) variants. The reference sequence NM_000092 of COL4A4 was used.

A novel heterozygous single nucleotide insertion, c.4760_4761insC and a previously reported likely pathogenic 18 bp deletion, c.1323_1340delTGGCTTGCCTGGAGCACC have been identified in the *COL4A4* gene in the index patient (Figure 3; Boye et al., 1998; Nabais Sá et al., 2015; Imafuku et al., 2018). The novel single nucleotide insertion (c.4760_4761insC) in exon 47 of *COL4A4* gene causes frameshift (p.Cys1588Metfs^∗^46) which finally results into the formation of truncated COL4A4 protein of 1634 amino acids instead of the wild type COL4A4 protein consisting of 1690 amino acids. Hence, it is a *“loss-of-function”* variant. Sanger sequencing confirmed that this variant was inherited in the index patient from her mother. This variant is not present in other unaffected family members as well as in 100 normal control individuals. This variant was not found in HDMD, ExAC or in 1000 Genome databases. According to the variant interpretation guidelines of the American College of Medical Genetics and Genomics (ACMG), this variant is classified as a *“likely pathogenic”* variant (Richards et al., 2015).

The previously reported 18-bp deletion (c.1323_1340delTGGCTTGCCTGGAGCACC) was also identified in the index patient (Boye et al., 1998; Nabais Sá et al., 2015; Imafuku et al., 2018). Sanger sequencing confirmed that this 18-bp deletion was inherited in the index patient from her father. This 18-bp deletion in exon 47 of the *COL4A4* gene causes *in-frame* deletion of Pro-Gly-Lys-Pro-Gly-Ala from Pro at 441. It is a likely pathogenic variant. This variant was not present in other unaffected family members as well as in the 100 ethnically matched unrelated normal control individuals.

These two variants were co-segregated well with the disease phenotype in this index patient. These two *loss-of-function* variants cause ARAS in this index patient with a compound heterozygosity.

## Discussion

Alport syndrome is a rare and hereditary renal disorder with extreme genotypic and phenotypic heterogeneity. AS generally starts with hematuria and proteinuria, which often results into end stage renal disease (ESRD). In normal individuals, glomerulus is functioning as a filtering unit of kidney. Physiologically, the most important part of the filtering system in the human kidney is the GBM. GBM is primarily composed by type IV collagen. The type IV collagen is encoded by three genes (*COL4A3, COL4A4,* and *COL4A5*), so variants in any of these genes lead to fragile and disorganized GBM. In patients with AS, GBM progressively becomes more fragile and disorganized which often results into end stage renal disease (ESRD). Although, no hearing impairment has been identified in the index patient, in later age she might experience AS associated hearing loss.

Here, we investigated a 24-year-old Chinese woman with Alport syndrome. Targeted next generation sequencing and Sanger sequencing revealed a novel single nucleotide insertion c.4760_4761insC and a previously reported likely pathogenic 18 bp deletion (c.1323_1340delTGGCTTGCCTGGAGCACC) in the *COL4A4* gene in the index patient (Boye et al., 1998; Nabais Sá et al., 2015; Imafuku et al., 2018). This novel insertion and likely pathogenic 18 bp deletion are inherited in the index patient from her mother and father, respectively. The findings also supported that these two *loss-of-function* variants causes AS in the index patient, with an autosomal recessive mode of inheritance by compound heterozygosity.

This 18 bp deletion (c.1323_1340delTGGCTTGCCTGGAGCACC) in the *COL4A4* gene has been previously reported in three cases with a compound heterozygosity (Boye et al., 1998; Nabais Sá et al., 2015; Imafuku et al., 2018). In all these three cases, the 18 bp *in frame* deletion causes ARAS along with other variants in *COL4A4* gene. All these reported patients were presented with only proteinuria and hematuria but without any FSGS like lesion. The individuals with only heterozygous 18 bp *in frame* deletion was reported with microhematuria (Gross et al., 2003). In our study, the index patient’s father was phenotypically normal though he was identified with this heterozygous 18 bp *in frame* deletion in *COL4A4* gene.

Human *COL4A4* gene is located at the long arm of Chromosome 2, comprising of 54 exons. *COL4A4* gene is encoding the α4 chain of type IV collagen. The α4 chain consists of 1690 amino acids. In addition, functionally, α4 chain is a very important part of forming the type IV collagen, which is the major component of GBM. Moreover, the α4 chain is used to show a tissue specific expression pattern in glomerulus, eye and inner ear (Nogueira et al., 2000). So, mutated *COL4A4* gene produces non-functional α4 chain which is unable to form the triple helix with α3 and α5 chains and finally disturb the structural stabilization of the type IV collagen which in turn leads to the formation of a fragile and disorganized GBM.

In the family considered in this study, no extrarenal manifestations or clinical symptoms have been identified. The index patient’s parents are asymptomatic. The index patient was primarily presented with proteinuria and hematuria. However, in this study, the index patient was identified with FSGS like lesion, which is in line with previous finings for ARAS patients with variants in the *COL4A4* gene (Malone et al., 2014). So, we can hypothesize that FSGS like lesion can be regarded as secondary manifestations upon the primary manifestations of defect in GBM associated with the formation of mutated or non-functional type IV collagen (van Paassen et al., 2004).

Presently, targeted next generation sequencing has become the most significant way for rapid and cost-effective screening, allowing us to perform easy and accurate clinical diagnosis of AS patients. Recent studies showed that the application of targeted exome sequencing for identification of genetic variations for the patients with complex renal phenotypes is useful (Chatterjee et al., 2013; Malone et al., 2014). In addition, the heterogeneity of symptoms in TBMN and other glomerular diseases could be explained by the role of unknown modifier genes (Voskarides et al., 2008a,b). The interaction between GBM and podocytes seems to play a key role for identifying major genetic modifiers which in turn leads to the development of novel drug target for glomerular diseases (Voskarides et al., 2007; Pierides et al., 2009). In this study, we selected a normal control group of 100 individuals. The number of control individuals though is not sufficient and represents one of the limitations in this study.

## Concluding Remarks

In conclusion, our present study identified two (one novel and one reported) heterozygous variants in *COL4A4* gene in an ARAS patient. The result of our study not only expands the COL4A4 variants spectrum but also showed the application of targeted next generation sequencing for easy, rapid and accurate clinical diagnosis based on genetic screening. Our study is the first to report on an ARAS patient that manifested with FSGS like lesion, harboring compound heterozygous variants in *COL4A4* gene.

## Ethics Statement

Family members of this three generation Chinese family have given written informed consent as they are participating in this study. The Ethical Committee of the Union Hospital, Tongji Medical College, Huazhong University of Science and Technology, Wuhan, China, reviewed and approved our study protocol in compliance with the Helsinki declaration.

## Author Contributions

JX conceived and supervised this study. FZ and WL performed the experiments. FZ, WL, and ZL analyzed the data. ZL and HZ wrote the manuscript. All authors read the final version of the manuscript.

## Conflict of Interest Statement

The authors declare that the research was conducted in the absence of any commercial or financial relationships that could be construed as a potential conflict of interest.
